# Molecular effects of the consumption of margarine and butter varying in *trans* fat composition: a parallel human intervention study

**DOI:** 10.1186/s12944-022-01675-1

**Published:** 2022-08-18

**Authors:** Dominik Guggisberg, Kathryn J. Burton-Pimentel, Barbara Walther, René Badertscher, Carola Blaser, Reto Portmann, Alexandra Schmid, Thomas Radtke, Hugo Saner, Nadine Fournier, Ueli Bütikofer, Guy Vergères

**Affiliations:** 1 Agroscope, Bern, Switzerland; 2 Preventive Cardiology and Sports Medicine, University Clinic for Cardiology, Inselspital, University of Bern, Bern, Switzerland; 3 Present addresses: Epidemiology, Biostatistics and Prevention Institute, University of Zurich, Zurich, Switzerland; 4 Present addresses: Institute for Social and Preventive Medicine, University of Bern, Bern, Switzerland; 5 Bioinformatics Core Facility, Swiss Institute of Bioinformatics, Lausanne, Switzerland

**Keywords:** Butter, Margarine, *Trans* fatty acids, Circulating lipids, Metabolome, Transcriptome

## Abstract

**Background:**

Whereas the dietary intake of industrial *trans* fatty acids (iTFA) has been specifically associated with inflammation, cardiovascular disease, and type 2 diabetes, understanding the impact of dietary fats on human health remains challenging owing to their complex composition and individual effects of their lipid components on metabolism. The aim of this study is to profile the composition of blood, measured by the fatty acid (FAs) profile and untargeted metabolome of serum and the transcriptome of blood cells, in order to identify molecular signatures that discriminate dietary fat intakes.

**Methods:**

In a parallel study, the molecular effects of consuming dairy fat containing ruminant TFA (rTFA) or margarine containing iTFA were investigated. Healthy volunteers (*n* = 42; 45–69 y) were randomly assigned to diets containing margarine without TFA as major source of fat (wTFA control group with 0.4 g TFA per 100 g margarine), margarine with iTFA (iTFA group with 4.1 g TFA per 100 g margarine), or butter with rTFA (rTFA group with 6.3 g TFA per 100 g butter) for 4 weeks. The amounts of test products were individually selected so that fat intake contributed to 30–33% of energy requirements and TFA in the rTFA and iTFA groups contributed to up to 2% of energy intake. Changes in fasting blood values of lipid profiles (GC with flame-ionization detection), metabolome profiles (LC-MS, GC-MS), and gene expression (microarray) were measured.

**Results:**

Eighteen FAs, as well as 242 additional features measured by LC-MS (185) and GC-MS (54) showed significantly different responses to the diets (P_FDR-adjusted_ < 0.05), mainly distinguishing butter from the margarine diets while gene expression was not differentially affected. The most abundant TFA in the butter, i.e. TFA containing (E)-octadec-11-enoic acid (C18:1 t11; *trans* vaccenic acid), and margarines, i.e. TFA containing (E)-octadec-9-enoic acid (C18:1 t9; elaidic acid) were reflected in the significantly different serum levels of TFAs measured after the dietary interventions.

**Conclusions:**

The untargeted serum metabolome differentiates margarine from butter intake although the identification of the discriminating features remains a bottleneck. The targeted serum FA profile provides detailed information on specific molecules differentiating not only butter from margarine intake but also diets with different content of iTFAs in margarine.

**Trial registration:**

ClinicalTrials.gov NCT00933322.

**Supplementary Information:**

The online version contains supplementary material available at 10.1186/s12944-022-01675-1.

## Introduction

*Trans* fatty acids (TFA) are fatty acids (FA) that have at least one double bond in *trans* configuration [1]. Historically, the major dietary source of TFA were partially hydrogenated fats, which were widely promoted to address obesity and cardiovascular disease (CVD) [2]. However, emergence of evidence that causally linked these fats with an increased risk of CVD [3–5], has led to widespread implementation of recommendations to limit consumption of partially hydrogenated oils, the majority stipulating that dietary TFA should be kept as low as possible [6, 7].

TFAs also occur naturally, in small quantities, in meat and dairy products, due to the action of bacterial enzymes that act in the rumen to bio-hydrogenate unsaturated FA. The TFA isomers from industrial (iTFA) and ruminant (rTFA) fats are chemically similar, although they vary in their relative concentrations [8]. Given the decline in iTFA intake, the relative importance of rTFA in the diet has increased, with recent estimates showing that rTFA was the major dietary source of TFAs (0.1 to 0.7% energy intake) for more than two-thirds of the countries surveyed [9].

The impact of rTFA on risk of CVD is still under debate [10]. Many epidemiological studies have demonstrated deleterious effects of iTFA but not rTFA on CVD risk [11–14], but others have reported equivalent effects of all TFA isomers on CVD risk factors [15, 16]. Furthermore, in a review of intervention studies, a positive relationship between all FAs with a double bond in *trans* configuration and plasma LDL to HDL cholesterol ratio was described [8]. Some inconsistencies in these findings can be attributed to the different quantities of TFAs consumed; indeed while positive associations between circulating lipoproteins and high intakes of rTFA or iTFA have been reported, these associations were lost at lower intakes of rTFA, as generally consumed in the diet [17, 18]. Nevertheless, a systematic review of randomised clinical trials found no evidence for an association between CVD risk factors and rTFA intakes of up to 4.2% energy intake [19].

In contrast to the wealth of observational data associating TFA with cardiovascular health, the understanding of molecular mechanisms mediating the effects of TFA on CVD risk is more limited [20]. However, there is evidence that TFA influences the regulation of multiple physiological processes [13], including hyperlipidemia [21], promotion of inflammation and cell death [22], reduced transforming growth factor-β (TGF-β) responsiveness [23], and endothelial dysfunction [13, 24]. Interestingly, preclinical studies comparing the major isoforms of rTFA and iTFA suggest that while these fats show some common behaviour, they can also act differentially depending on the molecular pathway assessed; for example rTFA may mediate its effects on cholesterol and FA synthesis via PPARs, while iTFA seems to specifically stimulate cholesterol synthesis via the activation of SREBP2-mediated gene regulation [20]. Thus, evaluation of the CVD risk of rTFA may require an understanding of the molecular effects of the different fats present in the food matrix.

Importantly, although dietary TFA are not consumed in isolation but with a complex mixture of associated FAs, few human studies have taken untargeted approaches in order to evaluate how these dietary fats modify the blood composition [25, 26]. Lipid profiling together with omics technologies (transcriptomics and metabolomics) could thus be a powerful approach for demonstrating the effects of dietary fats and their TFAs on molecular and metabolic functions and pathways. An earlier cross-over study measuring the FA profile of the milk of lactating women having ingested alpine butter or margarine during 10 days demonstrated that the milk FA profile of the study participants was modified specifically by the type of ingested fat [27]. Also, the blood FA profile was shown to associate with the adherence of young subjects to the Mediterranean diet [28]. In addition to FA profiling, serum metabolomics, although more exploratory in nature, might also identify molecules that are sensitive to the dietary intervention [29]. Finally, changes in the composition of the serum induced by the dietary interventions might induce a differential expression of genes in blood cells [30].

We previously reported the primary objectives of a parallel intervention study in which healthy subjects were allocated a diet containing up to 2% of daily energy intake from rTFA (alpine butter) or iTFA (margarine rich in iTFA) or a diet without TFA (margarine) for a duration of 4 weeks. These interventions did not show significant effects on brachial artery flow mediated dilation (primary objective) or on biomarkers of inflammation or coagulation. However, small but significant increases of total cholesterol and LDL-cholesterol were observed with the rTFA intervention (total cholesterol: rTFA group over wTFA group 1.04 (1.00 to 1.07 mmol/L); LDL-cholesterol: rTFA group over wTFA group 1.08 (1.03 to 1.14 mmol/L)) [31].

Although not clinically relevant these modest changes suggest that other molecular markers, which are sensitive to the dietary interventions, may be identified in the blood of the participants. The aim of the present work is therefore to make use of this study design for an exploratory analysis that investigates how the short-term intake of different sources of dietary fats differing in their composition (plant-based margarine vs animal-based butter) and content in TFA impact on the blood composition. To this end, the FA profile of blood (measured by GC-FID), the untargeted metabolome of serum (measured by GC-MS and LC-MS), and the blood cell transcriptome (measured by microarrays) were analysed and the results reported here.

## Methods

### Subjects

Participant recruitment, inclusion and exclusion criteria, randomization and compliance have been described in detail previously [31]. Briefly, a total of 142 healthy men and women, between 45 and 69 years were enrolled in the study. Exclusion criteria were known cardiovascular disease, smoking, hypertension, diabetes, abnormal liver and kidney function, existing signs of inflammation, anaemia, electrolyte abnormalities (Na, K, Ca), intake of medication (including supplements such as vitamins and minerals), and allergies to milk products. Of these, 125 participants were included in the per protocol analysis. Written informed consent for participation in the study was obtained from all participants and all procedures were conducted in accordance with the Declaration of Helsinki.

For the present analyses, we report on a predefined subgroup of 42 subjects selected from the main cohort using stratified sampling with respect to sex and treatment group (fourteen subjects per group). High-resolution FA analysis (GC-FID), gas chromatography mass spectrometry (GC-MS) and liquid chromatography mass spectrometry (LC-MS) analysis was completed for the entire subset while gene expression was assessed in whole blood of 21 randomly selected subjects from the same subset (seven subjects per intervention group).

### Study design and interventions

The study was a randomized, controlled, double-blind, three-arm, parallel-group intervention, designed to investigate the health effects of TFA intake from industrial and ruminant sources [31]. The study was approved by the cantonal ethical committee of Bern, Switzerland and registered at www.clinicaltrials.gov (NCT00933322). Block randomization with stratification for gender and age was used to assign subjects to one of three diets: a diet with alpine butter rich in ruminant TFA (rTFA group), a diet with margarine rich in industrial TFA (iTFA group) and a diet with margarine without TFA (wTFA group, control).

The study protocol began with a run-in period of 2 weeks, during which all study participants followed the wTFA diet, and was followed by a 4-week intervention period with the assigned diets (rTFA, iTFA, wTFA) according to the randomization [31]. All dietary fats were provided to the subjects in the form of individual amounts of the test fats (alpine butter with 6.3 g total TFA, without conjugated linoleic acid (CLA), per 100 g; margarine with 4.1 g total TFA per 100 g; margarine without TFA containing 0.4 g total TFA per 100 g) and 15–25 g/day rapeseed oil (to balance for essential FAs) in order for the fat intake to cover 33–36% of the individual energy requirement and for TFA to contribute to max 2% of total energy intake in the rTFA and iTFA groups.

During the whole study period the participants followed a prescribed diet according to Swiss nutrition recommendations. The participants received detailed instructions about the study diet from a dietician and were followed at regular intervals during the study. Daily energy requirement was individually calculated. The participants were allowed to consume fat-free products on an individual basis according to the recommendations provided by the dietician. Throughout the study, participants were required to fill in a daily food diary. The proportion of fat was individually calculated and had to be consumed in form of the study products. Details of the dietary restrictions and evaluation of dietary adherence are published elsewhere [31]. At each visit, weight was assessed and BMI calculated.

### Blood sampling

Blood samples were collected from the antecubital vein at baseline (after the 2-week run-in period) and at the end of the intervention period (week 6) after an overnight fast. The preparation and processing of blood samples to assess clinical biochemistry and biomarkers of inflammation, coagulation and endothelium function have been described previously [31]. For GC-MS and LC-MS analyses, serum was separated and stored in microtubes at − 80 °C until assayed. Whole blood was collected for microarray analysis in PAXgene blood RNA tubes and frozen at − 20 °C for 24 h before transfer to − 80 °C until further treatment.

### High-resolution lipid analysis with GC-FID

Serum samples were prepared for analysis by addition of 15 μL of internal standard (C13:0, 7.5 μg/15 μL) to 100 μL of serum, followed by methylation of free FAs with MeOH/HCl (25 °C for 45 min). A post-reaction treatment for neutralization was applied with Na_2_CO_3_ and extraction was performed with 300 μL hexane. Samples were measured by gas chromatography with flame ionization detector (GC-FID) (6890, Agilent Technologies, Santa Clara, CA) equipped with a CP-Sil 88 fused-silica capillary column (length 100 m, i.d. 0.25 mm, stationary-phase film thickness 0.20 μm). The carrier gas was hydrogen (1.5 ml/min). The on-column injector was hold in “Track Oven Mode”. The injector temperature was always 3 °C higher than the oven temperature. The detector temperature was 225 °C. The oven temperature was held constant for 5 min at 60 °C, then increased to 165 °C at 14 °C/min, held at 165 °C for 1 min, increased again to 225 °C at 2 °C/min, held at 225 °C for 17 min, increased again to 240 °C at 10 °C/min, and was finally held at 240 °C for 5 min. The injection volumes was 0.5 μL as described previously [32]. Butter and margarine samples (*n* = 2) were assayed previously by Radtke et al. [31], using the same GC-FID technique as for serum samples with sample preparation according to the method of Collomb and Bühler [32]. The data was reprocessed and reintegrated here (Table 2) with some additional reference lipids included. Eighty-five features (66 single FAs and 19 sum parameters) were quantitatively analysed in the serum and dietary fats. The FA isomers that cannot be separated by the chromatographic steps are indicated by the plus sign “+” joining various cis (c) and/or trans (t) forms of the FAs.

A control fat made from butter was used for peak assignment. In addition, a control chart with a human serum standard from Sigma-Aldrich (Human serum from human male AB plasma, H4522) was routinely run with the test samples. For each new measurement series the response factors were determined with the following fatty acid methyl esters (all from Sigma-Aldrich): Methyl butyrate (C4, C_5_H_10_O_2_; Methyl valerate (C5, C_6_H_12_O_2_); Methyl hexanoate (C6, C_7_H_14_O_2_); Methyl octanoate (C8, C_9_H_18_O_2_); Methyl nonanoate (C9, C_10_H_20_O_2_); Methyl decanoate (C_10_, C_11_H_22_O_2_); Methyl undecanoate (C11, C_12_H_24_O_2_); Methyl dodecanoate (C12, C_13_H_26_O_2_); Methyl tridecanoate (C13, C_14_H_28_O_2_); Methyl myristate (C14, C_15_H_30_O_2_); Methyl palmitate (C16, C_17_H_34_O_2_); Methyl stearate (C18, C_19_H_38_O_2_); Methyl oleate, (C18:1c9, C_19_H_36_O_2_).

### Transcriptomic analysis and data pre-processing

Total RNA was extracted from whole blood samples and treated to deplete for globin mRNA according to the protocol described by Gille et al. [33]. Whole genome transcript profiling was then performed with HG-U219 oligonucleotide expression probe arrays (Affymetrix, USA). Preparation of samples for profiling including reverse transcription, amplification, amplified RNA labelling, purification, fragmentation, and hybridization steps were performed as defined previously [33]. Arrays were measured in accordance with manufacturer’s recommendations using Affymetrix GeneAtlas™System. Raw array data was imported to the R environment (version 4.1.2) [34] and corrected for background noise, log2 transformed, normalized for inter-array variation (quantile normalization) [35] and summarized using the *rma* (Robust Multichip Average) function from affy (version 1.72.0) [36]. In addition, probe sets were filtered to keep only the most variable probe set per gene (based on standard deviation), probe sets assigned to gene symbols (hgu219.db, version 3.2.3) [37] and probe sets with average expression (log2) > 5. Of the 49′386 probe sets, 6′128 probe sets were retained for further analysis.

### Untargeted GC-MS analysis and data pre-processing

Sample preparation for the untargeted analysis of the serum samples by GC-MS was based on the method published by Trimigno et al. [38] using a GC-MS 7890B/MS5977A (Agilent Technologies, Santa Clara, CA, USA) with a CombiPAL autosampler (CTC-Analytics AG, Zwingen, Switzerland). After deconvolution, features from subjects that only appeared in less than three samples were eliminated (*n* = 4828 remaining features). A second manual integration was conducted on 54 selected features that showed significant differences between treatment group responses to the interventions (see *Statistical Analyses*). The areas were normalized with the isotopically-labelled fructose. The features demonstrating a significant treatment effect were searched in EI-Mass Spectral Library (NIST 2017, Gaithersburg, MD 20899–6410, USA), masslib-library (www.masslib.com). The molecules demonstrating sufficient potential for identification were acquired from Sigma-Aldrich (Buchs, Switzerland) and analyzed by GC-MS.

### Untargeted LC-MS analysis and data pre-processing

Serum samples were thawed, treated, and analyzed according to the protocol described by Pimentel et al. [29], using a maXis 4G+ (Bruker, Bremen, Germany) coupled to a 3000 RS UHPLC (Thermo, Basel, Switzerland). Raw data (m/z values, retention times and intensities) were imported into Progenesis QI (V2.4) (Waters, Switzerland) for retention time alignment, peak detection, sample loading normalization, deconvolution of masses (including detection of adducts), and final export of peak volumes for further statistical treatment. The pre-processed dataset (~ 85′000 features) was imported into R (version 3.5.2) [39] and filtered in two steps. All features that showed a mean in QC samples (*n* = 21) that were < 4 times higher than the mean for the final three blanks were excluded. In addition, features with a relative standard deviation > 30% in the QC samples were excluded to leave a reduced dataset of 11′616 features. Identification was carried out by a mass search in the Human Metabolome Database (HMDB v4.0 2018 [40]) and NIST (National Institute of Standards and Technology Database, V14). Successive identification was then confirmed by LC-MS where exact mass and retention time of the metabolites were compared with standards. The identification was differentiated at four levels [41–43]. Metabolites identified by comparison with a standard based on their mass (±10 ppm) and retention time (± 10%), e.g. two independent and orthogonal data, are classified as level 1. Metabolites showing at least 80% match to a theoretical MSMS spectral library but without available standard are assigned level 2. Level 3 is attributed to a compound class (e.g. if multiple similar standards match the retention time and the mass spectrum). Finally, level 4 is attributed to unidentified compounds that are nonetheless well separated from other molecules and those peak surface can be integrated.

### Statistical analyses

#### Data exploration

All statistical analyses were performed in the R environment (version 3.5.2) [39]. The normality of baseline clinical characteristics and metabolomics datasets was explored through Shapiro-Wilk tests of normality and visually by quantile-quantile plots. Principal components analysis (PCA) was used with all datasets to visually inspect sources of variation, in particular to exclude the presence of batch effects, outliers, or potential confounders (e.g. sex) (lipids and metabolomics datasets: ropls package, v1.4.4, 2016, [44]; transcriptomics dataset: stat function ‘prcomp’ visualized with ggplot2 (version 3.3.5) [45].

#### Clinical data

Non-parametric univariate statistics were used for the current analysis in view of the reduced study population size and multiple features that were not distributed normally. Baseline clinical characteristics, blood biochemistry, inflammation and endothelium biomarkers of the study population were compared by treatment group using the Kruskal-Wallis test (*P* < 0.05). Pairwise comparison of results were performed with Conover-Iman test (*P* < 0.05).

#### GC-FID lipid analysis

The Kruskal Wallis test was used to compare the changes in lipid profiles in response to the three interventions. For significant results, a post hoc Wilcoxon signed-rank test was completed to compare each with adjustment of *P*-values for multiple testing using the Benjamini-Hochberg FDR method [46] (significance where P_FDR-adjusted_ < 0.05). The sum parameters for the classes of FAs were integrated into this analysis. The response to the intervention for these analyses was defined for each participant as the delta change between measures at endpoint (week 6) and baseline (week 2). Features showing a significant difference between the three treatments were presented in heatmaps using the “Metabolomics” package (v0.1.4) [47] with distance calculated by “*Canberra*” method and clustering using the “*ward. D2*” method [48].

#### Gene and pathway analysis

The molecular effects of the dietary interventions were investigated by assessing the change in gene expression in whole blood (i.e. delta change between baseline and post-treatment), with delta values calculated using log_2_-transformed data, thus interpretable as log_2_-fold expression changes (log_2_FC). Differentially expressed genes for each group and between groups were identified by performing a paired moderated t-test on the responses to the intervention using R package Limma (Linear Models for Microarray Data) (version 3.50.0) (P_FDR-adjusted_ < 0.05) [46].

Gene set enrichment analysis (GSEA) [49] was used to explore the results of the differential analyses. GSEA determines whether a predefined set of genes show statistically significant, consistent differences between two biological states by calculation of an enrichment score (ES) for each geneset. A ‘normalized’ ES (NES) is calculated to account for differences in number of genes in each geneset. GSEA was carried out for a selection of genesets (*n* = 20) from the Hallmark human reference geneset collection (mSigDB, Broad Institute, version 7.5.1) that describe pathways that are regulated by TFA, including lipid and cholesterol metabolism, inflammation, apoptosis, autophagy, coagulation, adipose tissue regulation, and oxidative stress [20]. Significance of enrichments were confirmed by comparing the ES to those obtained by random permutations (*n* = 1000 iterations) of the ranked gene with FDR correction for multiple testing (significance where P_FDR-adjusted_ < 0.05) [46].

#### Untargeted metabolomics analysis

The same statistical workflow as described above for the GC-FID FA analysis was applied for the untargeted LC-MS metabolomics dataset to identify molecular biomarkers that were differentially modulated by the interventions, (comparing delta change between baseline and post-treatment). For the untargeted GC-MS dataset, a two-step statistical analysis was applied to select putative compounds that could discriminate the different effects of the interventions (delta change between baseline and post-treatment) for the reintegration step of data processing. In the first step, univariate analyses were applied to identify metabolites showing a different response between the dietary treatments using the Kruskal-Wallis test (*P* < 0.05). This was complemented with the identification of further discriminating metabolites from orthogonal partial least squares - discriminant analysis (OPLS-DA) models using the Ropls package (v1.4.4, 2016) [44] to compare the effects of each pair of treatments (for valid models, the top 100 metabolites were selected for reintegration). After reintegration, the second step of analysis applied a Wilcoxon signed-rank test to compare the responses of the reintegrated metabolites to the treatment in pairwise assessments (P_FDR-adjusted_ < 0.05).

#### Correlation analyses

Correlation analyses were conducted to assess the relationships between biomarkers measured previously [31], including blood biochemistry (glycemia, insulinemia, blood lipids) and biomarkers of inflammation and endothelium function and the FAs measured by targeted GC-FID analysis. Spearman’s correlation test was used to associate the biomarker and FA changes in response to the dietary interventions (delta change between baseline and post-treatment) with visualization by corrplot (v0.84) [50] with significance where P_FDR-adjusted_ < 0.05 [46]. This analysis was restricted to the fourteen clinical parameters that showed no significant difference between groups at baseline. The effect of the different dietary intervention on these associations was considered by repeating the analysis with Spearman’s partial correlation test to control for the effect of diet (ppcor Package, v 1.1) [51].

## Results

### Subject characteristics and clinical chemistry

The 42 participants selected for the nutrigenomic analyses showed similar baseline clinical characteristics to the main cohort [31] (Table 1). There were significant differences between the treatment groups for baseline levels of insulin, interleukin-6 (IL-6), tumor necrosis factor (TNF) receptors (1 and 2), and endothelial leucocyte adhesion molecule (ELAM-1). Therefore, these markers were not considered further.

**Table 1:** Baseline characteristics of subjects. Assessment after the run-in phase (week 2)

	wTFA group	iTFA group	rTFA group	*P*
	(*n* = 14)	(*n* = 14)	(*n* = 14)	
Age (years)	56.5 (52.3, 58.8)	52.0 (46.8, 58)	51.0 (47, 56.5)	0.373
Sex (% male)	50	50	50	
BMI (kg/m^2^)	25.20 (21.0, 26.8)	24.60 (22.5, 28.2)	25.35 (23.8, 27.4)	0.778
Glucose (mmol/L)	4.68 (4.5, 5.0)	5.10 (4.7, 5.4)	4.73 (4.4, 5.0)	0.260
Insulin (mU/L)	5.00 (3.3, 6.3)	5.75 (3.5, 7.9)	3.65 (1.9, 4.4)	0.040*
Total cholesterol (mmol/L)	5.035 (4.6, 5.3)	4.895 (4.0, 5.6)	4.465 (4.3, 5.0)	0.338
HDL-C (mmol/L)	1.49 (1.3, 1.8)	1.36 (1.1, 1.5)	1.33 (1.2, 1.7)	0.401
LDL-C (mmol/L)	3.22 (2.8, 3.7)	3.06 (2.6, 3.8)	2.67 (2.5, 3.3)	0.193
Ox-LDL Ab (mU/ml)	254 (162, 476)	483 (347, 1394)	811 (411, 1167)	0.092
Triglycerides (mmol/L)	0.87 (0.75, 1.0)	0.89 (0.62, 1.4)	0.66 (0.58, 0.92)	0.314
Lp-a (mg/L)	0.13 (0.11, 0.35)	0.20 (0.08, 0.47)	0.12 (0.04, 0.8)	0.933
Apo A1 (g/L)	2.61 (1.3, 3.4)	2.05 (1.5, 2.4)	2.14 (1.7, 2.8)	0.457
Apo B (g/L)	0.87 (0.29, 1.23)	0.78 (0.41, 1.02)	1.05 (0.86, 1.39)	0.156
hs-CRP (mg/L)	0.6 (0.24, 1.49)	0.96 (0.7, 1.98)	0.82 (0.5, 2.09)	0.371
IL-6 (pg/ml)	0.74 (0.65, 0.98)	0.7 (0.6, 0.96)	1.09 (0.97, 1.38)	0.027*
TNFα (pg/ml)	2.40 (2, 5)	3.37 (1, 5.2)	3.81 (1.8, 7.2)	0.701
TNF-Receptor 1 (pg/ml)	798 (705, 974)	995 (941, 1103)	1197 (997, 1395)	0.007**
TNF-Receptor 2 (pg/ml)	2636 (2290, 4089)	2664 (2457, 3406)	1748 (1461, 2098)	0.033*
ELAM-1 (ng/ml)	2.5 (1.6, 3.4)	2.6 (1.2, 4.8)	5.5 (3.9, 8.2)	0.007**
Endothelin (pg/ml)	1.9 (1.6, 2.7)	2 (1.7, 2.3)	1.7 (1.3)	0.638
ICAM (ng/ml)	17.5 (10, 32.4)	19.1 (10.9, 32.2)	25.8 (20.7, 29.7)	0.274
VCAM (ng/ml)	212 (124, 320)	150 (113, 241)	286 (183, 415)	0.099

Legend: Data are presented as medians and IQ1, IQ3 between brackets. Kruskall-Wallis test was used to determine significant difference between subjects in the three groups. *P* < 0.05: *, *P* < 0.01: **, *P* < 0.001: ***

*Abbreviations*: *Apo A1* Apolipoprotein A1, *Apo B* Apolipoprotein B, *hs-CRP* High-sensitivity C reactive protein, *ELAM-1* Endothelial leucocyte adhesion molecule, *HDL-C* High-density lipoprotein cholesterol, *ICAM* Intercellular adhesion molecule, *iTFA* Diet enriched with industrial trans fatty acids, IL-6 Interleukin-6, *LDL-C* Low-density lipoprotein cholesterol, *Lp-a* Lipoprotein a, *ox-LDL Ab* Antibodies of oxidized low-density lipoprotein, *rTFA* Diet enriched with ruminant trans fatty acids, *TNF* Tumor necrosis factor, *VCAM* Vascular cellular adhesion molecule, *wTFA* Diet without trans fatty acids

### Altered lipid profiles in serum reflect dietary fat composition

The total fat content of the three test products were very similar but differences were present for specific FAs (Table 2). The butter contained comparable, although higher, levels of total TFAs to the margarine with iTFA, and it was confirmed that the margarine without TFA (wTFA) contained only traces of TFA. However, the relative composition of the most abundant TFAs differed between the iTFA margarine and the butter; the TFA in the iTFA margarine contained both (E)-octadec-12-enoic acid (C18:1 t12) (0.3%) and the mixture of (E)-octadec-10-enoic acid and (E)-octadec-11-enoic acid (C18:1 t10 + t11) (1.4%), whereas the TFA in rTFA in butter was mainly C18:1 t10 + t11 (3.5%). In addition, the butter contained higher levels of SFA, in particular hexadecanoic acid (C16:0) and octadecanoic acid (C18:0), than the margarines despite lower levels of dodecanoic acid (C12:0). Total MUFA and total PUFAs were present at higher levels in both margarines than the butter due to higher levels of (Z)-octadec-9-enoic acid (C18:1c9) and (9Z,12Z)-octadeca-9,12-dienoic acid (C18:2c9c12), respectively.

**Table 2:** Composition of the most important fatty acids of the butter and the two margarines with and without TFA (g 100 g^−1^ product), used in this study. The repeatability (r) is based on the analysis of 35 different milk samples measured in duplicate according to the method of Collomb and Bühler [32] (Agroscope, unpublished internal report 13.8.ME.028)

Experimental fat (g/100 g product)	Repeatability	Margarine without TFA (wTFA)	Margarine with TFA (iTFA)	Butter (rTFA)
Total fat content		83.4	84.6	85.1
**SFA**				
C4:0 (butanoic acid)	0.327	–	–	2.6
C6:0 (hexanoic acid)	0.089	0.2	0.2	1.5
C8:0 (octanoic acid)	0.074	2.1	2.1	0.8
C10:0 (decanoic acid)	0.111	1.5	1.6	1.7
C12:0 (dodecanoic acid)	0.146	11.4	11.9	1.9
C14:0 (tetradecanoic acid)	0.385	4.8	4.9	7.3
C15:0 (pentadecanoic acid)	0.081	–	–	1
C16:0 (hexadecanoic acid)	0.943	18.1	16.2	19.6
C17:0 (heptadecanoic acid)	0.176	–	–	0.4
C18:0 (octadecanoic acid)	0.330	3.4	4.2	8.5
C20:0 (icosanoic acid)	0.014	0.2	0.2	0.1
C 22:0 (docosanoic acid)	0.021	–	–	0.1
13Me-C15:0 (13-methyltetradecanoic acid)	0.019	–	–	0.3
12Me-C15:0 (12-methyltetradecanoic acid)	n.d.	–	–	0.5
14Me-C16:0 (14-methylpentadecanoic acid)	0.042	–	–	0.3
15Me-C17:0 (15-methylhexadecanoic acid)	0.010	–	–	0.3
14Me-C17:0 (14-methylhexadecanoic acid)	0.029	–	–	0.3
16Me-C18:0 (16-methylheptadecanoic acid)	n.d.	–	–	0.1
Total SFA	n.d.	41.7	41.5	47
**MUFA** ***cis***				
C14:1c9 ((Z)-tetradec-9-enoic acid)	0.076	–	–	0.6
C16:1c9 ((Z)-hexadec-9-enoic acid)	0.087	0.1	0.1	1.1
C18:1c9 ((Z)-octadec-9-enoic acid)	0.665	24.4	23.9	15.2
C18:1c11 ((Z)-octadec-11-enoic acid)	0.027	0.8	0.9	0.7
C18:1c12 ((Z)-octadec-12-enoic acid)	0.083	–	0.2	0.2
C18:1c13 ((Z)-octadec-13-enoic acid)	0.027	–	0.1	0.1
C20:1c8 + c9 ((Z)-icos-8-enoic acid + (Z)-icos-9-enoic acid)	n.d.	–	–	0.1
C20:1c11 ((Z)-icos-11-enoic acid)	0.015	0.1	0.1	–
Total MUFA cis		25.4	25.3	18
**PUFA** ***cis***				
C18:2c9c12 ((9Z,12Z)-octadeca-9,12-dienoic acid)	0.087	5	4.6	1.2
C18:3c9c12c15 ((9Z,12Z,15Z)-octadeca-9,12,15-trienoic acid)	0.056	1.1	1.2	1
C20:4c ((5Z,8Z,11Z,14Z)-icosa-5,8,11,14-tetraenoic acid)	0.020	–	–	0.1
C20:5c ((5Z,8Z,11Z,14Z,17Z)-icosa-5,8,11,14,17-pentaenoic acid)	0.009	–	–	0.1
C22:5c ((7Z,10Z,13Z,16Z,19Z)-docosa-7,10,13,16,19-pentaenoic acid)	0.011	–	–	0.1
Total PUFA cis		6.1	5.8	2.5
**TFA**				
C16:1 t9 ((E)-hexadec-9-enoic acid)	0.029	–	–	0.2
C18:1 t6 + t7 + t8 + t9 ((E)-octadecenoic acid mix_t6 + t7 + t8 + t9_)	0.090	0.1	1.8	0.2
C18:1 t10 + t11 ((E)-octadecenoic acid mix_t10 + t11_)	0.245	0.1	1.4	3.5
C18:1 t12 ((E)-octadec-12-enoic acid)	0.115	–	0.3	0.3
C18:1 t13 + c6 + c7 (octadecenoic acid mix_t13 + c6 + c7_)	0.196	–	0.4	0.5
C18:1u (octadecenoic acid u)	n.d.	–	0.1	0.3
C18:2u (octadecadienoic acid u)	n.d.	0.1	0.1	0.2
C18:2t9c12 ((9E,12Z)-octadeca-9,12-dienoic acid)	n.d.	0.1	–	0.5
C18:2c9t11 ((9Z,11E)-octadeca-9,11-dienoic acid)	0.077	–	–	1.5
Total trans (without CLA)		0.4	4.1	6.3
**Sum parameters (cumulative values)**				
Total C18:1 t		0.2	4	5
Total C18:2 t (with CLA)		0.1	0.1	2.7
Total C18:2 t (without CLA)		0.1	0.1	1.1
Total CLA		–	–	1.6
Total TFA (without CLA)		0.4	4.1	6.3
Total TFA (with CLA)		0.4	4.1	7.9
Total ω-3 FA		1.2	1.2	1.7
Total ω-6 FA		5	5.3	2

Legend: - = concentration < 0.01 g

*Abbreviations*: *u* Unknown, *mix*_*position*_ Mixture of different isomers, *CLA* Conjugated linoleic acids, *iTFA* Industrial *trans* fatty acids, *MUFA* Monounsaturated fatty acids, *PUFA* Polyunsaturated fatty acids, *rTFA* Ruminant *trans* fatty acids, *SFA* Saturated fatty acids, *TFA Trans* fatty acids, *wTFA* Without *trans* fatty acids. The FAs isomers that cannot be separated by the chromatographic steps are indicated by the plus sign “+” joining various cis (c) and/or trans (t) forms of the FAs

**Fig. 1:**
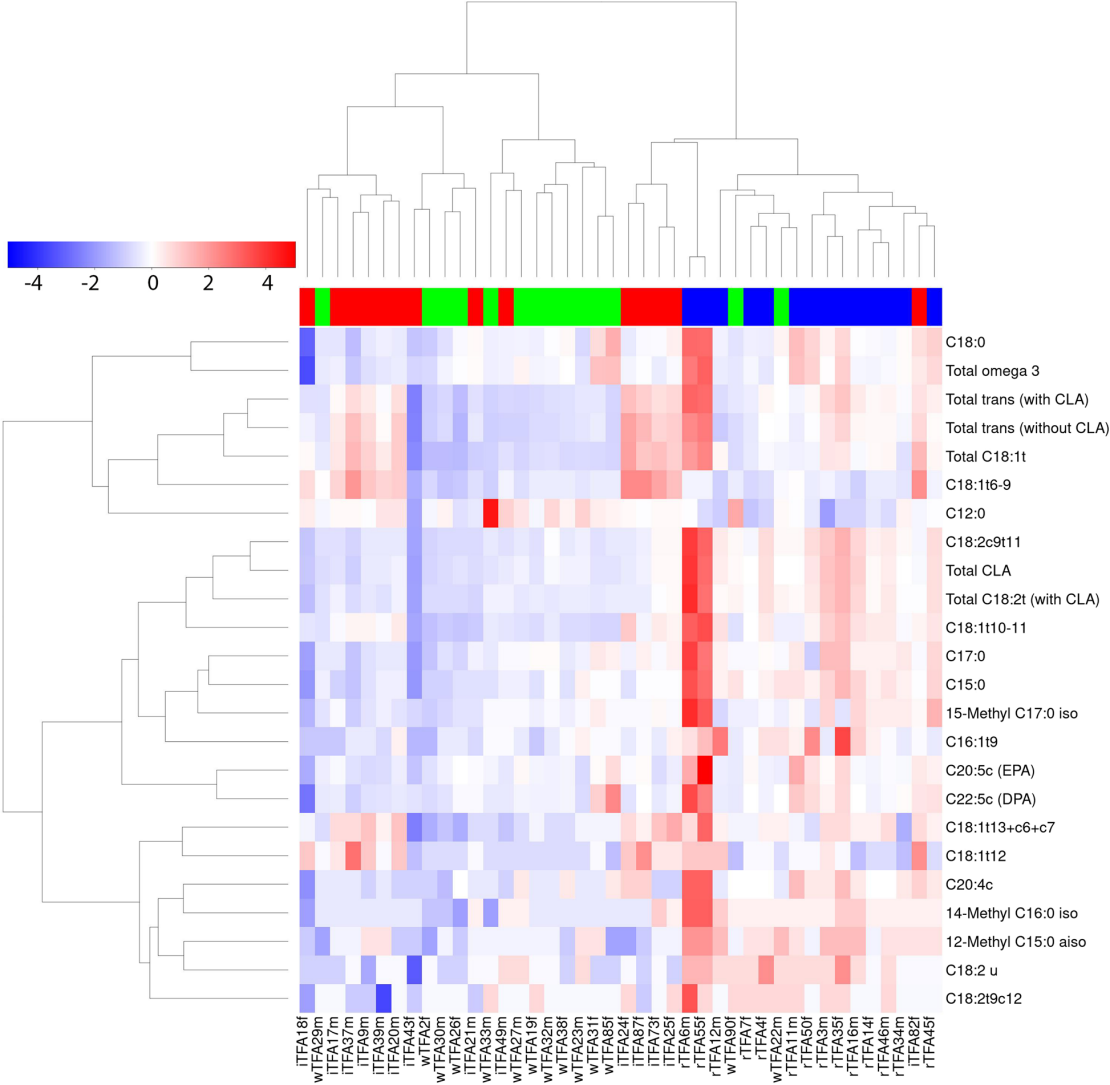
Heatmap of 24 lipid parameters (18 lipids and 6 sum parameters) measured by high-resolution GC-FID that were selected from 85 features (66 single FAs and 19 sum parameters) by a Kruskal-Wallis test (P_adjusted FDR_ < 0.05). The response of each subject (delta change between baseline and post-treatment) is denoted in columns with annotation for treatment groups, rTFA group (blue), margarine wTFA (green) and margarine with iTFA (red), study number and sex (m = male, f = female)

Out of 85 features (i.e. 66 FAs and 19 sum parameters), a total of 18 FAs (four long-chain SFAs, three branched-chain FAs, three PUFAs and all TFAs) as well as six sum parameters showed significantly different responses for at least one intervention (P_FDR-adjusted_ < 0.05) (Table 3). A heatmap of the 24 FAs (inclusive of six sum parameters) that showed significantly different responses to the treatment is presented in Fig. 1, showing clustering of the 42 subjects into the three treatment groups (iTFA, wTFA and rTFA) with six exceptions.

**Table 3:** Delta change in serum levels of lipids [μg mL^−1^ serum] after 4 week treatments. Only lipids that change significantly differently between treatments are shown. The Spearman’s correlation between median serum change in lipid per treatment group and content of lipids in the corresponding food products is represented by a letter code: +: weak positive (rho < 0.6); ++ moderate positive (rho 0.7–0.9); +++: strong positive (rho > 0.9). All correlations are positive

Fatty Acids	Median wTFA	IQR wTFA (Q1, Q3)	Median iTFA	IQR iTFA (Q1, Q3)	Median rTFA	IQR rTFA (Q1, Q3)	P_FDR-adjusted_^#^	sp
[μg mL^−1^]	(*n* = 14)		(*n* = 14)		(*n* = 14)			
**SFA**								
C12:0	10^b^	−15, 45	0^b^	−10, 15	-60^a^	−70, − 17.5	**	+
C15:0	0^a^	−10, 10	-5^a^	−17.5, 7.5	30^b^	20, 40	***	++
C17:0	5^a^	−10, 17.5	-5^a^	−17.5, 10	35^b^	30, 82.5	**	++
C18:0	30^a^	− 368, 583	-125^a^	−320, 300	660^b^	190, 1783	*	+
12Me-C15:0	0^a^	10, 0	0^a^	−10, 0	10^b^	10, 20	**	++
14Me-C16:0	0^a^	−7.5, 10	0^a^	0, 10	10^b^	10, 20	**	++
15Me-C17:0	0^a^	−7.5, 10	5^a^	−10, 10	30^b^	2.5, 50	*	++
**PUFA cis**								
C20:4c	0^a^	0, 10	-5^a^	−10, 0	20^b^	10, 35	**	++
C20:5c	15^a^	0, 42.5	-10^a^	−5, 37.5	65^b^	30, 153	**	++
C22:5c	10^a^	−10, 27.5	-10^a^	−20, 15	60^b^	32.5, 128	***	++
**TFA**								
C16:1 t9	0^a^	0, 10	0^a^	−10, 10	25^b^	10, 47.5	**	++
C18:1 t6 + t7 + t8 + t9	10^a^	−17.5, 10	115^b^	92.5, 185	10^a^	−7.5, 20	**	+++
C18:1 t10 + t11	0^a^	−10, 10	75^b^	27.5, 90	115^c^	95, 153	***	+++
C18:1 t12	0^a^	0, 0	25^b^	12.5, 30	10^b^	0, 17.5	**	++
C18:1 t13 + c6 + c7	0^a^	0, 10	30^b^	12.5, 38	20^b^	12.5, 30	**	+
C18:2u	0^a^	−10, 7.5	0^a^	−10, 0	10^b^	10, 17.5	**	++
C18:2 t9 + c12	0^b^	0, 7.5	-5^a^	−10, 0	10^b^	0, 10	**	+++
C18:2c9 + t11	0^a^	−10, 7.5	0^a^	−17.5, 7.5	80^b^	52.5, 105	**	++
**Sum parameters**								
Total C18:1 t	10^a^	−7.5, 27.5	285^b^	163, 335	155^b^	103, 198	***	+
Total C18:2 t (with CLA)	-5^a^	−17.5, 0	0^a^	−27.5, 10	80^b^	40, 123	***	++
Total CLA	−10^a^	-10, 10	0^a^	−17.5, 10	75^b^	45, 115	***	++
Total TFA (without CLA)	20^a^	−7.5, 47.5	275^b^	115, 328	165^b^	125, 225	***	+
Total TFA (with CLA)	20^a^	−10, 62.5	290^b^	77.5, 338	225^b^	172.5, 337.5	***	+
Total ω-3 FA	90^ab^	−40, 228	-15^a^	−200, 135	260^b^	102.5, 908	*	++

Legend: ^#^Kruskal wallis test results (P_FDR-adjusted_ < 0.05: *, P_FDR-adjusted_ < 0.01: **, P_FDR-adjusted_ < 0.001: ***). Different letters (a, b, c) denote significant differences between treatment based on paired Wilcoxon signed-rank test (P_FDR-adjusted_ < 0.05)

*Abbreviation*: *sp* Spearman correlation, *IQR* Interquartile range. The FAs isomers that cannot be separated by the chromatographic steps are indicated by the plus sign “+” joining various cis (c) and/or trans (t) forms of the FAs

C18:1 t10 + t11 and C18:1 t12 were the major TFAs found in the test products. The quantified levels of these FAs in the test products (margarines and butter) are shown in Table 2, together with their relative changes in serum after the dietary interventions (Table 3). Whereas serum levels of C18:1 t10 + t11 and C18:1 t12 were both increased in the iTFA group (C18:1 t12 being significantly higher than C18:1 t10 + t11), only C18:1 t10 + t11 was increased in the rTFA group. Neither TFA was significantly modified in the wTFA group.

Total cholesterol was significantly (*P* < 0.05) increased in the rTFA group (0.44 mmol/L; Q1: -0.24, Q3; 0.48) compared to the wTFA group (0.02 mmol/L; Q1: 0.04, Q3; 0.63). The results of the iTFA group (0.17 mmol/L; Q1: -0.33, Q3; 0.20) were not significantly (*P* > 0.05) different from the two other groups. LDL cholesterol was significantly (P < 0.05) increased in the rTFA group (0.37 mmol/L; Q1: -0.10, Q3; 0.35) compared to the wTFA group (− 0.03 mmol/L; Q1: 0.18, Q3; 0.53) and the iTFA group (0.01 mmol/L; Q1: -0.24, Q3; 0.25). HDL cholesterol was not significantly (*P* > 0.05) increased in the rTFA group (− 0.01 mmol/L; Q1: -0.16, Q3; 0.10) compared to the wTFA group (− 0.05 mmol/L; Q1: -0.18, Q3; 0.04) and the iTFA group (0.01 mmol/L; Q1: -0.24, Q3; 0.25). Triglycerides were not significantly (*P* > 0.05) increased in the rTFA group (0.04 mmol/L; Q1: -0.04, Q3; 0.22) compared to the wTFA group (0.04 mmol/L; Q1: -0.08, Q3; 0.14) and the iTFA group (0.06 mmol/L; Q1: -0.11, Q3; 0.18).

### Associations between circulating lipids and clinical markers

**Fig. 2:**
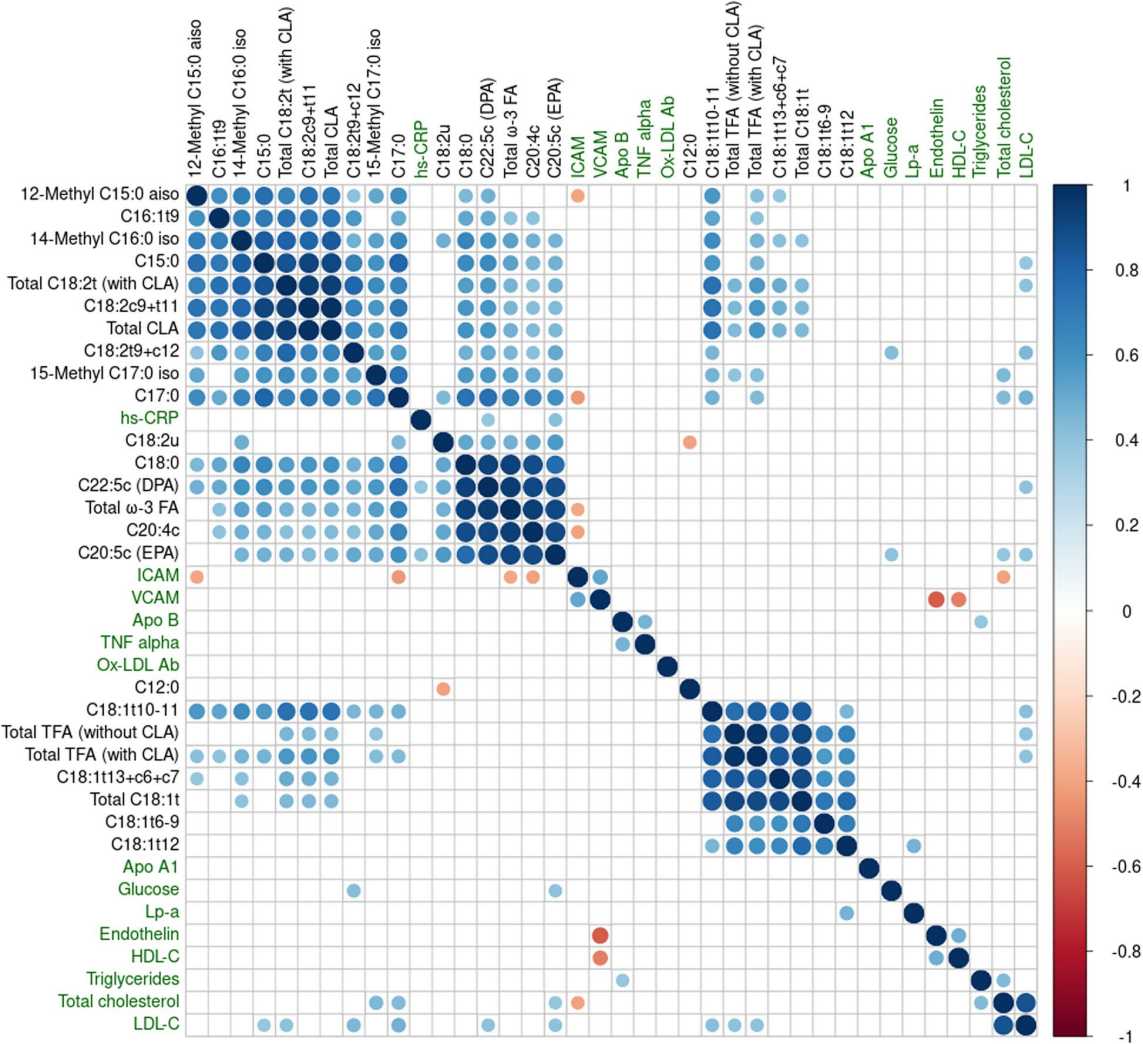
Spearman correlation analysis in the whole study-population identified some positive (blue) and negative (red) significant associations between blood lipids showing a significant response to the interventions (*n* = 24) and clinical parameters (*n* = 14) after the 4 week treatments (delta change between baseline and post-treatment). Significance was considered where P_adjusted FDR_ < 0.05. Non-significant correlations are left blank. The clustering method was Ward D2. The size of the coloured symbols and the coloured scale to the right indicate the rho correlation value. Clinical parameters highlighted in green

An overview of the significant correlations found between changes in plasma free FAs and clinical biomarkers after the dietary interventions is presented in Fig. 2. The strongest correlations were observed between FAs. Most of the significant associations were between lipids or between the clinical biomarkers as these two categories of parameters were ranked separately with the exception of C12:0. Also, the FAs had stronger correlations coefficients than the clinical biomarkers. Finally, most of the correlations were positive, in particular for the FAs. Nevertheless, some significant associations between targeted FAs and clinical biomarkers were identified, as detailed in Supplement Table 1. Multiple positive correlations were observed for the FAs and LDL-cholesterol including dairy-associated biomarkers, C15:0 and C17:0, several PUFAs, and individual TFAs, namely C18:1 t10 + t11 and (9E,12Z)-octadeca-9,12-dienoic acid (C18:2 t9 + c12)). All of these correlations remained significant after adjusting for the treatment effect. Several FAs that were associated with LDL-cholesterol were also positively associated with changes in total cholesterol. Conversely, HDL-cholesterol was not significantly associated with any FAs. Some FAs were positively associated with blood glycemia, hsCRP, intercellular adhesion molecule, and lipoprotein a, but these associations were generally not significant after controlling for the effect of treatment with the exception of C17:0 that was inversely associated with inter cellular adhesion molecule ICAM. No significant associations were observed between the selected lipids and triglycerides, apolipoprotein B, endothelin, TNF-α, and vascular cellular adhesion molecule (VCAM). In addition, no significant associations with clinical biomarkers were observed for the dominant TFA species in iTFA, C18:1 t6 + t7 + t8 + t9.

### Limited effects of dietary fats on gene expression in blood

Overall, 496 genes were differentially expressed between baseline (week 2) and endpoint (week 6) in at least one of the three intervention groups (*P* < 0.05) but the responses were not statistically significant nor did they differ significantly between the intervention groups (P_FDR-adjusted_ > 0.05). GSEA pathway enrichment did not reveal any pathway of the 20 genesets tested that was significantly differentially enriched whether for individual treatments or when comparing treatments.

### Biomarkers of butter revealed in untargeted metabolomics analyses of serum

#### LC-MS untargeted metabolomics

**Fig. 3:**
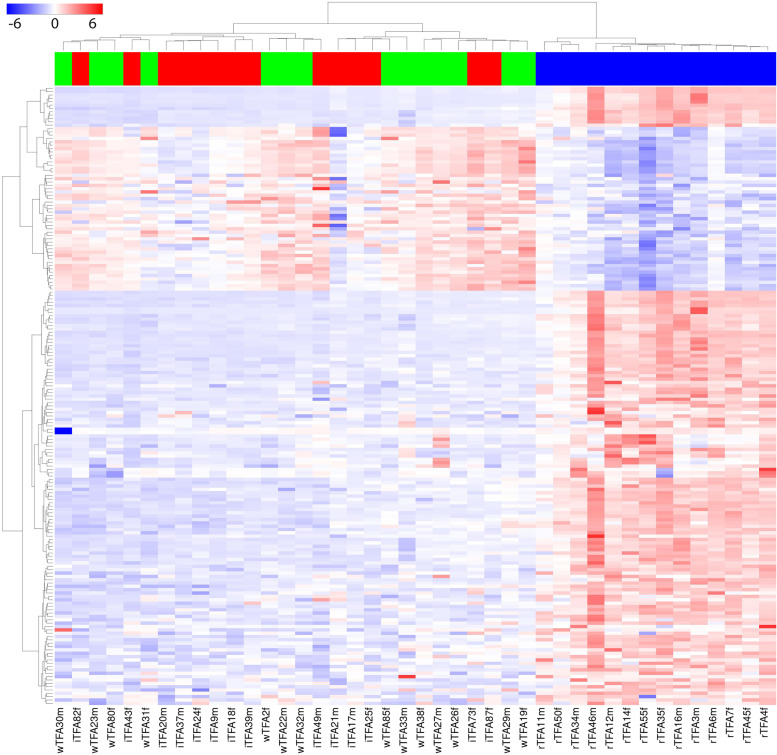
Heatmap of 185 LC-MS features (rows) that were selected from 11′616 (normalised) LC-MS features by a Kruskal-Wallis test (P_adjusted FDR_ < 0.05). The response of each subject (delta change between baseline and post-treatment) is denoted in columns with annotation for treatment groups, rTFA group (blue), margarine wTFA (green) and margarine with iTFA (red), study number and sex

**Fig. 4:**
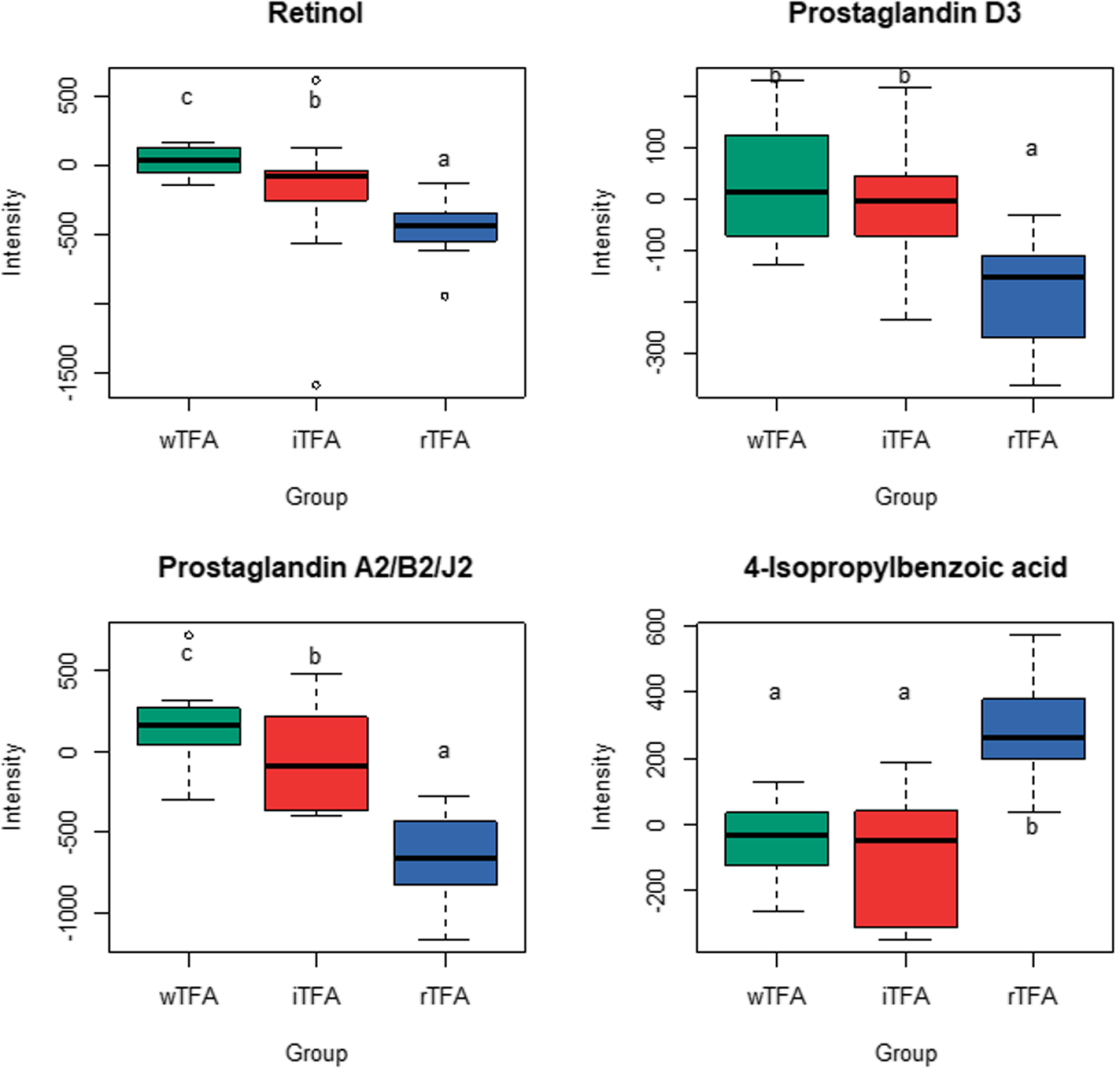
Boxplots of LC-MS metabolites that show a significant response (delta change between baseline and post-treatment) to the diets grouped by those that are either increased or decreased in the rTFA group. The identification levels of these metabolites were 1 for retinol and prostaglandin D3 and 3 for the two others. Different letters indicate significantly different values (paired Wilcoxon signed-rank Test, P_FDR-adjusted_ < 0.05) paired. Plots show the IQR (box), the median dividing the IQR (—), with dashed line whiskers that extend to the last point included in the 1.5 x IQR range and outliers outside this range identified (o). One outlier in wTFA boxplot for 4-isopropylbenzoic acid was eliminated (baseline: 475.6, endpoint: 6464.7). This subject was however not removed from the robust statistical analysis

A total of 185 features measured in blood by LC-MS showed significantly different responses to the three interventions (P_FDR-adjusted_ < 0.05). These metabolites are presented in a heatmap (Fig. 3) which shows almost exclusively positive values, i.e. increased concentrations at the end of the 4-week intervention, as well as a separation of the butter group from the margarine groups while the margarine groups (wTFA and iTFA) were not differentiated. Of the 185 significant features, two were identified at level 1 (Fig. 4, Supplement Table S2). Of the identified metabolites, three showed a relative decrease in the rTFA group, relative to the wTFA and/or iTFA groups, including the fat-soluble vitamin, retinol. An inspection of the levels of these metabolites at the end of the run-in phase showed they were present at similar levels between the three treatment groups while after the butter treatment the levels of retinol and prostaglandins decreased (Supplement Fig. S1). 4-isopropylbenzoic acid showed an increase after the rTFA intervention relative to the wTFA and iTFA groups.

#### GC-MS untargeted metabolomics

**Fig. 5:**
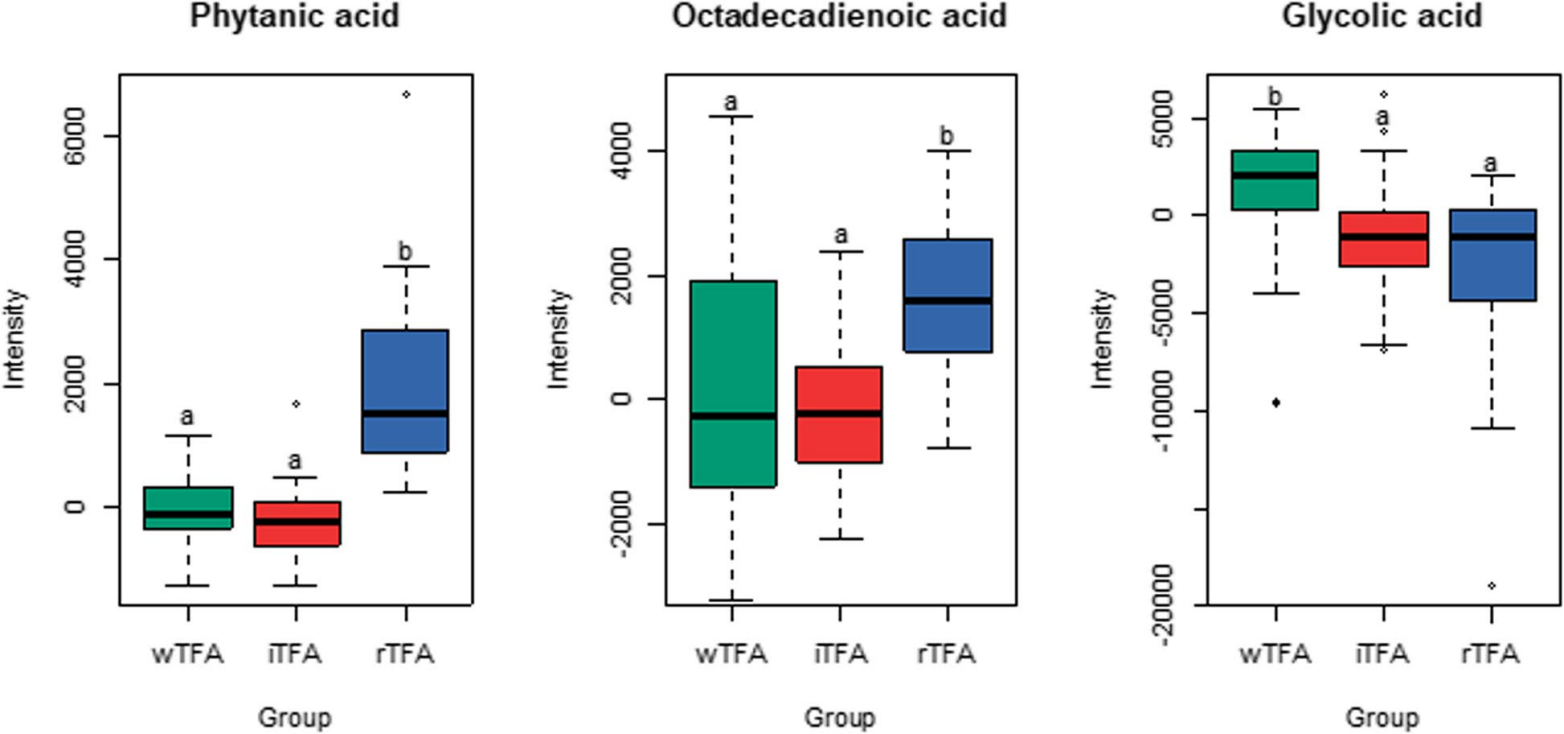
Boxplots of identified GC-MS metabolites that show different responses (delta change between baseline and post-treatment) to the diets. Different letters indicate significantly different values (paired Wilcoxon signed-rank Test, P_FDR-adjusted_ < 0.05) paired. Plots show the IQR (box), the median dividing the IQR (—), with dashed line whiskers that extend to the last point included in the 1.5 x IQR range and outliers outside this range identified (◊)

The GC-MS analysis revealed three identified metabolites that showed significantly different responses to the treatments after manual integration of the signals (P_FDR-adjusted_ < 0.05) (Fig. 5). Phytanic acid was clearly increased in butter treatment (*P* < 0.001) compared to wTFA and iTFA. An isomer of octadecadienonic acid (C18:2), was also significantly increased in the rTFA group, though it was present both in butter and margarine (*P* = 0.007). This molecule was classified at the level 3 of identification because of its unknown isomeric structure. Finally, glycolic acid, an α-hydroxy acid, was significantly decreased in rTFA (*P* = 0.037), relative to the wTFA group.

## Discussion

The present study investigated the molecular and metabolic impact of 4 weeks butter compared to margarine intake (with and without TFA) on fasting blood using a targeted analysis of free FA (GC-FID), transcriptomic analysis of whole blood gene expression and two untargeted metabolomic approaches (LC-MS and GC-MS).

### Lipid composition of dietary interventions is reflected in serum of healthy adults

Reanalysis of the data on serum lipids (total cholesterol, LDL-C, HDL-C, TG) in the subcohort of 42 participants selected for this study showed similar effects of the dietary fat interventions than already reported for the original whole cohort of 129 participants [31]. In particular, a small but significant increase in total cholesterol and LDL-cholesterol was observed in the group with the butter diet compared to the groups with the margarine diets. This reanalysis indicates that the sub-cohort of 42 participants is representative of the larger cohort with regard to lipid metabolism. The profiling of the lipids present in the intervention diets in serum before and after dietary interventions revealed a distinct FA signature of the intervention diets in serum. For those FAs that responded differently to the interventions, remarkable consensus could be observed between the relative change in serum and the different quantity of lipids in the products. Indeed, with the exception of total TFA that included CLA, the relative changes in serum lipids directly reflected the differences in the product composition, even for those lipids that were present in relatively low levels such as the PUFAs. We where indeed able to demonstrate this close relationship between composition of TFA in different dietary fats and circulating levels of TFA after exposure to diets with differing but still relatively low TFA content.

Among the SFA that discriminated between rTFA and iTFA diets, C15:0 and C17:0 were both specifically increased after rTFA, confirming their previously described role as dietary biomarkers of dairy intake [52, 53]. Several other odd-chain methylated FA were also raised specifically after dairy intake including 15-methylhexadecanoic acid (15Me-C17:0), which has previously been associated with butter intake [54].

The PUFAs (5Z,8Z,11Z,14Z)-icosa-5,8,11,14-tetraenoic acid (C20:4c; arachidonic acid), (5Z,8Z,11Z,14Z,17Z)-icosa-5,8,11,14,17-pentaenoic acid (C20:5c; EPA) and (7Z,10Z,13Z,16Z,19Z)-docosa-7,10,13,16,19-pentaenoic acid (C22:5c; DPA) were significantly increased after the rTFA diet, despite their presence at very low levels in butter (albeit higher than the margarines). C18:2c9c12 (linoleic acid), a dietary precursor of eicosanoids, was actually more abundant in the margarine diets although no significant differences in this lipid was observed in serum. The serum differences in eicosanoids therefore do not seem to be explained by the dietary composition of lipids but may rather reflect differences in metabolism of (9Z,12Z,15Z)-octadeca-9,12,15-trienoic acid (C18:3c9c12c15; linolenic acid) and C18:2c9c12 (linoleic acid). Indeed, there is some evidence to suggest that certain TFAs can disrupt the metabolism of essential PUFAs [55]. A change in the metabolism of PUFAs could not be confirmed in our transcriptomic analysis of blood cells. As the synthesis of PUFAs is localised in the liver, blood cell transcription might not have captured such tissue-specific regulation, particularly in the fasting state. Another limitation in using fasting samples is that changes in FAs that are rapidly metabolised may not be visible. Indeed, the absence of diet-specific differences for some short-chain SFAs and MUFAs despite differences in their presence in the composition of the diets may be attributable to rapid metabolism of these fats.

### Different TFAs in serum distinguish diets rich in rTFA compared to iTFA

Both diets containing TFA (iTFA and rTFA) induced similar increases in total TFA in serum that were significantly higher than the control diet without TFA (wTFA). Conversely, several TFAs showed high specificity to the source of TFA both in the product content and the serum after exposure to the diet. As expected, conjugated C18:2 FAs (CLA) were specifically associated with the rTFA diet due to their well-known production by rumen bacteria [56]. Interestingly, diet-related changes in TFAs were found for FAs present in relatively low quantities in the diet such as C16:1 t and C18:2 t isomers. The most abundant TFAs in the rTFA and iTFA diets, respectively C18:1 t6 + t7 + t8 + t9 and C18:1 t10 + t11, were both specifically elevated in serum after the corresponding diet. The chromatographic steps used to analyse the FAs cannot separate these mixtures of TFAs. However, (E)-octadec-9-enoic acid (C18:1 t9; elaidic acid) and (E)-octadec-11-enoic acid (C18:1 t11; trans vaccenic acid) are the major TFA in margarine and butter, respectively [57], so that these molecules likely contribute the most to the total content in these mixtures. C18:1 t9 (elaidic acid) has already been identified as a putative dietary intake biomarker of margarine and hardened vegetable fats, as like other TFAs it cannot be synthesized by the body [58]. Conversely, the major isomer of rTFA, C18:1 t11 (*trans* vaccenic acid) [59], is also present in dietary sources of iTFA, though at lower levels, as reflected by lower but significant increases in serum levels for the iTFA group.

### Associations of C18:1 t9 (elaidic acid) and C18:1 t11 (*trans* vaccenic acid) to cholesterol metabolism

In our study we also explored the relationship between circulating lipids following the intervention diet and various biomarkers of molecular pathways that can be influenced by TFAs, including inflammation, lipid/cholesterol metabolism, and coagulation. Our correlation data confirmed a positive association between various TFAs that were specifically elevated after the rTFA intervention, in particular C18:1 t10 + t11, including C18:1 t11 (*trans* vaccenic acid), and the small but significant increase in LDL-cholesterol after rTFA, which was described previously for the full dataset [31]. It is however important to note the similar associations observed for other FAs that were specifically elevated after rTFA including the dairy biomarkers C15:0 and C17:0. Thus the correlations might be interpreted as a collective effect of the dairy matrix on the LDL-cholesterol rather than a specific TFA-modulation of cholesterol [60]. No associations were found between C18:1 t6 + t7 + t8 + t9, including C18:1 t9 (elaidic acid) and the circulating biomarkers of inflammation and lipid metabolism. While serum C18:1 t9 (elaidic acid) has been associated with CVD outcomes Oshita et al. [61], the iTFA contained relatively low levels of TFAs and serum levels of the FAs in this short-term intervention remained consistently low, which could explain the lack of effect of the intervention on surrogate markers of cholesterol metabolism.

Transcriptomics and metabolomics approaches are methods sensitive to detect early biological responses and were selected for this study to help assess the effects of the relatively low levels of TFAs in the intervention diets. However, our transcriptomics data showed limited associations with the dietary interventions, which concurred with the previously reported absence of effects of the high intake of iTFA and rTFA for 3 weeks on the plasma proteome of healthy men [62]. Although blood transcriptomics can reflect systemic transcription changes in response to dietary interventions with fats, in particular under postprandial conditions [63], in this healthy population and the relatively low level of TFAs used in the diets, it was unfortunately not possible to confirm a distinct effect of the diets on the fasting transcriptome. Furthermore, only one type of metabolite was shown to distinguish between the three diets, a prostaglandin (A2/B2/J2), which was decreased after both TFA-containing diets but to a greater extent after rTFA, while prostaglandin D3 was only decreased after butter. Prostaglandins are regulatory compounds derived from dietary FAs, in particular arachidonic acid, that play important roles in many physiologic processes on many human organ systems, including inflammation [64]. Interestingly C18:1 t9 (elaidic acid) and C18:1 t11 (*trans* vaccenic acid) differently impact prostaglandins production in endothelial cells [65]. Although the prostaglandins tentatively identified in this report have hardly been reported in humans, our results suggest that plasma prostaglandins might be sensitive markers to assess the impact of dietary fats on inflammation in humans. However in the absence of responses in other metabolites or inflammatory biomarkers, this result should be interpreted with caution.

In previous studies, TFA intake has been associated with the inflammatory markers CRP and IL6, particularly in populations predisposed to metabolic illness [66, 67]. In our study with healthy subjects, no correlation between C18:1 t6 + t7 + t8 + t9, including C18:1 t9 (elaidic acid) or C18:1 t10 + t11, including C18:1 t11 (*trans* vaccenic acid), and the two inflammation markers were found. Similarly, Motard-Belanger et al. found no increase of CRP at the end of 4 weeks of rTFA or iTFA in a nutritional intervention, compared to the control [16]. It is noteworthy that both diets comprise relatively low but comparable levels of TFA (2% total energy intake), a little over the WHO public health guidelines which could explain the limited clinical effects of the diet previously reported by Radtke et al. [31].

### Biomarkers of dietary intake

The majority of metabolites that responded differently to the dietary interventions differentiated the butter effect from the similar effects of the two margarines. Several of the identified metabolites were increased in the rTFA group and might be considered as biomarkers of ruminant animal fat intake. These included 4-isopropyl-benzoic acid and FAs, octadecadienoic acid and phytanic acid. The branched-chain fatty acid (BCFA) phytanic acid is a degradation product from chlorophyll that is found in ruminant animal fat and has been proposed a putative biomarker of dairy fat [52, 68]. Some health benefits from phytanic acid intake have been described [69], such as prevention of metabolic syndrome or type 2 diabetes although dietary reduction of phytanic acid was also recommended for the management of infantile Refsum disease, one of the less severe of Zellweger spectrum disorders [70]. 4-isopropyl-benzoic acid (cuminic acid) is present in the seeds of *Cuminum cyminum* (cumin) (https://foodb.ca/compounds/FDB013929). Although its presence in foods, in particular dairy products, has not been reported it is interesting to note that *Cuminum cyminum* is used as a feed additive in livestock. The untargeted GC-MS methodology used to characterize octadecadienoic acid is not specific enough to differentiate the various C18:2 molecules that can possibly be present. The reader is thus referred to the GC-FID results for further details.

Dietary biomarkers of margarine could also be inferred by considering metabolites showing a relative reduction in the rTFA group relative to the baseline, which was the end of the run-in with the wTFA margarine. These included retinol, which although present in butter is supplemented, often at high levels, in margarines. Of note, dihydrophylloquinone, a hydrogenated form of vitamin K1 not naturally present in vegetable oils, has previously been reported as a candidate biomarker of TFA-intake from partially hydrogenated fat [71] but was not found with our untargeted LC-MS method.

For most of the molecules described above, the changes in their serum levels during the treatment phase are most likely attributed to the test products rather than to other factors such as endogenous metabolism or the intake of other food components independent of the intervention. Excluding these alternative explanations for the observed associations of the identified metabolites with the dietary intervention could be more precisely addressed by a postprandial analysis of serum in controlled acute intervention studies using butter and margarine as nutritional challenges.

### Strengths and limitations of study

A strength of this study was the use of different techniques to investigate the molecular effects of the dietary interventions under study. In particular, by using a targeted FA panel, we were able to discriminate between the circulating changes in specific FAs and TFAs that were present in the dietary sources of TFAs. This characteristics is important in the context of the distinct biological roles of these lipids. Using different metabolomics approaches in this study, we were also able to consider the wider impact of the diets on metabolites other than lipids.

Although the clustering of LC-MS data was more efficient than the targeted FA analytics to separate the intake of butter from the intake of margarine, we were limited by the challenge of identifying discriminating metabolites. A range of issues associated with the identification of the molecular features derived from metabolomics studies contributes to this bottleneck, including the quality of the databases, the stability of the laboratory metabolomics instrumentation (columns, detector …), and the availability of standards. Each of these parameters would need to be improved to increase the success rate of the identification step. The original study defined its number of participants based on the primary endpoint, brachial artery flow mediated dilation. In light of the extensive work load needed to conduct FA analysis the number of subjects was limited to 42 and this study was thus conducted on an exploratory basis. This number of subjects was, indeed, sufficient to identify differentiating metabolites, as confirmed with a recent studies investigating FA blood profiles in a similar number of subjects as a function of dietary adherence to the Mediterranean diet [28]. Our study was further limited by the restricted number of samples evaluated for whole blood transcriptomics, which did not capture the expected changes in lipid metabolism following the change in dietary fats. Although a previous study has reported changes in the fasting blood cell transcriptome of seven subjects as a result of a 2-week dairy intervention [30], our gene expression study remain exploratory: an higher number of subjects might have provided stronger insights into the reasons for the lack of changes in gene expression (i.e. insufficient statistical power vs real biological lack of response). However, gene expression remains a sensitive method to identify subtle metabolic change resulting from dietary interventions. Given that none of the 496 differentially expressed genes identified in this study retained statistical significance after correction for multiple testing, a targeted strategy specifically focusing on a small set of pre-defined genes with relevant function might have been more appropriate.

## Conclusion

The application of a combination of targeted and untargeted metabolomics to serum samples of humans supported the identification of distinct chemical signatures associated with the different dietary fats. We confirm that the different types and abundance of TFA isomers in rTFA and iTFA diets results in distinct changes in serum TFAs after 4 weeks of these diets. In particular, C18:1 t6 + t7 + t8 + t9, including C18:1 t9 (elaidic acid) and C18:1 t10 + t11, including C18:1 t11 (*trans* vaccenic acid) are differentially modulated by the TFA diets in concordance with their relative presence in the dietary fats. The associations of the different TFAs with clinical biomarkers did not seem specific to the TFAs but was rather related to the overall lipid composition of the dietary fats. Beyond the lipid analysis, we did not find clear biomarkers that distinguished the rTFA from iTFA perhaps reflecting the relatively low dose of TFAs used that did not result in clinically different phenotypes in the larger cohort of this study [31]. However, we did identify biomarkers that distinguished butter from the margarines including some putative exogenous markers, suggesting that this approach is useful for discriminating metabolic effects of similar foods.

## Supplementary Information

**Additional file 1: Supplement Figure S1.** Individual changes in identified LC metabolites during study intervention. Individual line plots coloured by intervention groups: wTFA (green), iTFA (red), rTFA (blue). One outlier in wTFA lineplot for 4-isopropylbenzoic acid was eliminated (baseline: 475.6, endpoint: 6464.7). This subject was however not removed from the robust statistical analysis. **Supplement Table S1.** Spearman correlations between fatty acids that responded differently to the interventions and clinical parameters using delta values. Significance for P_adjusted FDR_ < 0.05. **Supplement Table 2.** Identification of LC-MS metabolites.

## Data Availability

The transcriptome and metabolome datasets generated and analysed during the current study are available from the corresponding author on reasonable request.

## Abbreviations

CLA: Conjugated linoleic acid
CRP: C-reactive protein
GC: Gas-chromatography
GC-FID: GC with flame ionization detector
GSEA: Geneset enrichment analyses
HDL-C: High-density lipoprotein cholesterol
hs-CRP: High-sensitivity C-reactive protein
ICAM: Intercellular adhesion molecule
iTFA: Industrial trans fatty acids
IL-6: Interleukin-6
LC: Liquid-chromatography
LDL-C: Low-density lipoprotein cholesterol
MS: Mass spectrometry
MUFA: Monounsaturated fatty acids
OPLS-DA: Orthogonal partial least squares – discriminant analysis
PUFA: Polyunsaturated fatty acids
rTFA: Ruminant trans fatty acids
SFA: Saturated fatty acids
TFA: Trans fatty acids
TNF: Tumor necrosis factor
VCAM: Vascular cellular adhesion molecule
wTFA: Without trans fatty acids
