# Supplementary material for: Molecular effects of the consumption of margarine and butter varying in trans fat composition: a parallel human intervention study
Source: Lipids Health Dis. 2022 Aug 18;21:74. doi: 10.1186/s12944-022-01675-1 (PMC9389665; doi:10.1186/s12944-022-01675-1)
Supplement: Supplementary file 1 — Additional file 1: Supplement Figure S1. Individual changes in identified LC metabolites during study intervention. Individual line plots coloured by intervention groups: wTFA (green), iTFA (red), rTFA (blue). One outlier in wTFA lineplot for 4-isopropylbenzoic acid was eliminated (baseline: 475.6, endpoint: 6464.7). This subject was however not removed from the robust statistical analysis. Supplement Table S1. Spearman correlations between fatty acids that responded differently to the interventions and clinical parameters using delta values. Significance for Padjusted FDR < 0.05. Supplement Table 2. Identification of LC-MS metabolites. [file 12944_2022_1675_MOESM1_ESM.docx]

**Supplementary information – Guggisberg et al.**

**Supplement Figure S1.** Individual changes in identified LC metabolites during study intervention. Individual line plots coloured by intervention groups: wTFA (green), iTFA (red), rTFA (blue). One outlier in wTFA lineplot for 4-isopropylbenzoic acid was eliminated (baseline: 475.6, endpoint: 6464.7). This subject was however not removed from the robust statistical analysis.


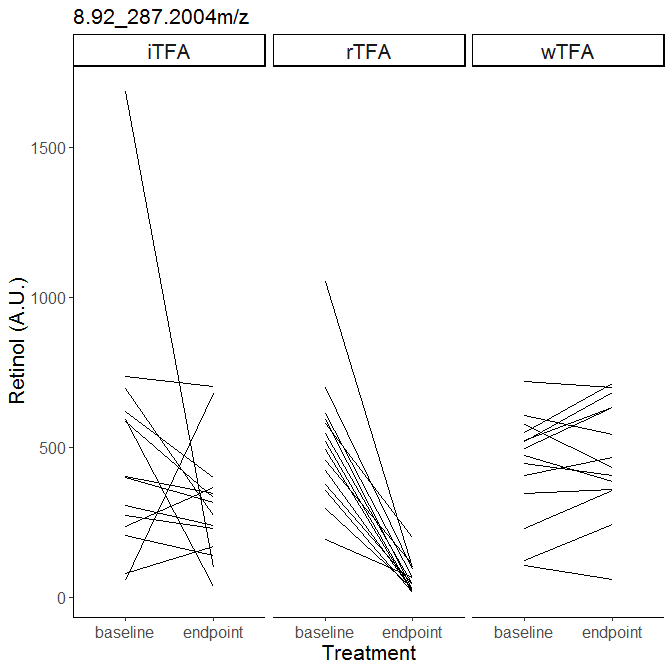


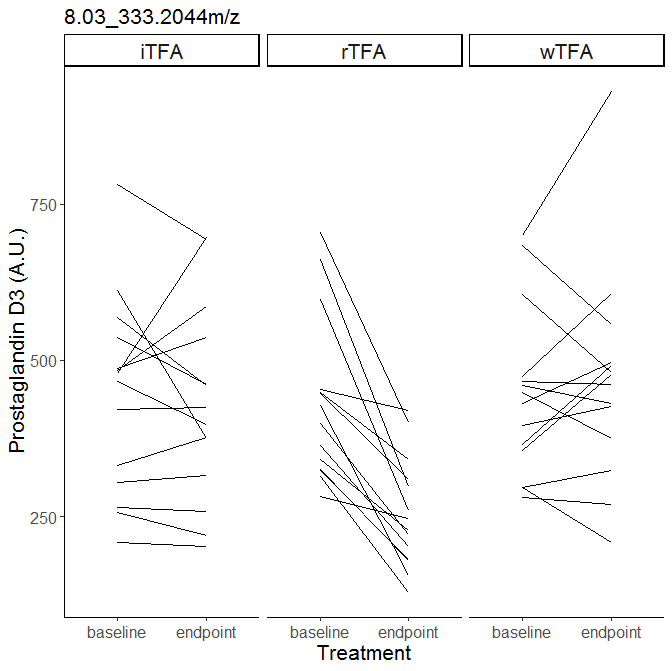


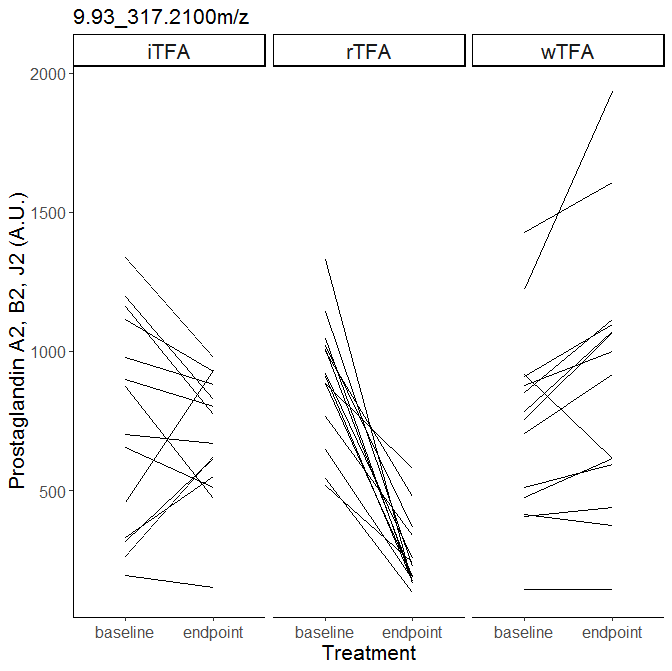


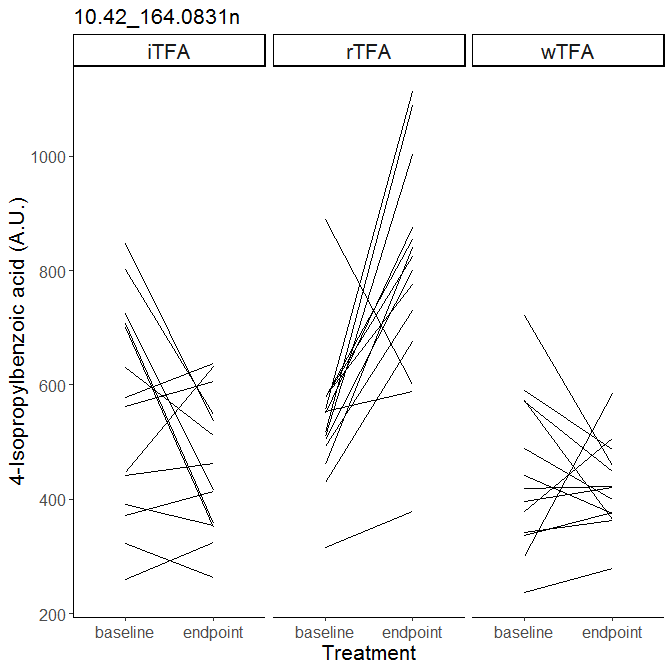


**Supplement Table S1** Spearman correlations between fatty acids that responded differently to the interventions and clinical parameters using delta values. Significance for P_adjusted FDR_ <0.05.

| FA | Glucose | Total cholesterol | LDL-cholesterol | hs-CRP | ICAM | Lp-a |
| --- | --- | --- | --- | --- | --- | --- |
| C12:0 |  |  |  |  |  |  |
| C15:0 |  |  | 0.387* |  |  |  |
| C17:0 |  | 0.438* | 0.471* |  | -0.432* |  |
| C18:0 |  |  |  |  |  |  |
| 12-Methyl-C15:0 |  |  |  |  | -0.395 |  |
| 14-Methyl-C16:0 |  |  |  |  |  |  |
| 15-Methyl-C17:0 |  | 0.449* |  |  |  |  |
| C20:4c |  |  |  |  | -0.403 |  |
| C20:5c | 0.403 | 0.388* | 0.405* | 0.411 |  |  |
| C22:5c |  |  | 0.401* | 0.385 |  |  |
| C16:1t9 |  |  |  |  |  |  |
| C18:1t6-9 |  |  |  |  |  |  |
| C18:1t10-11 |  |  | 0.417* |  |  |  |
| C18:1t12 |  |  |  |  |  | 0.463 |
| C18:1t13+c6+c7 |  |  |  |  |  |  |
| C18:2u |  |  |  |  |  |  |
| C18:2t9+c12 | 0.43 |  | 0.441* |  |  |  |
| C18:2c9+t11 |  |  |  |  |  |  |
| Total C18:1t |  |  |  |  |  |  |
| Total C18:2t (with CLA) |  |  | 0.407* |  |  |  |
| Total CLA |  |  |  |  |  |  |
| Total trans (without CLA) |  |  | 0.407* |  |  |  |
| Total trans (with CLA) |  |  | 0.401* |  |  |  |
| Total omega 3 |  |  |  |  | -0.384 |  |

No significant associations were found for the following clinical markers: triglycerides, HDL cholesterol, Apo A1, Apo B, ox-LDL Ab, endothelin, TNF-α, VCAM. ^*^ Correlation remained significant (P_adjusted FDR_<0.05) after correcting for treatment effect. Abbreviations: Apo A1, apolipoprotein A1; Apo B, apolipoprotein B;CLA, conjugated linoleic acid; hs-CRP, high-sensitivity C reactive protein; HDL-C, high-density lipoprotein cholesterol; ICAM, intercellular adhesion molecule; iTFA, diet enriched with industrial trans fatty acids; LDL-C, low-density lipoprotein cholesterol; Lp-a, lipoprotein a; rTFA, diet enriched with ruminant trans fatty acids; ox-LDL Ab, antibodies of oxidized low-density lipoprotein; TNF, tumor necrosis factor; VCAM, vascular cellular adhesion molecule; u, unknown configuration of cis/trans

wTFA: diet without trans fatty acids.

**Supplement Table 2** Identification of LC-MS metabolites.

| Identified metabolites: | RT in Study [min] | RT for standard [min] | Mass in Study | Mass for standard | Mass error [ppm] | Identification level | Supplier |
| --- | --- | --- | --- | --- | --- | --- | --- |
| Retinol | 8.92 | 9.2 | 287.2004 m/z | 287.2000 m/z | -1.39 | 1 | Sigma-Aldrich  (Buchs, Switzerland) |
| Prostaglandin D3 | 8.03 | 8.9 | 333.204 m/z (-H_2_O) | 333.206 m/z (-H_2_O) | 6.002 | 1 | Adipogen (Liestal, Switzerland) |
| 4-Isopropylbenzoic acid | 10.42 | 10.3 | 165.090 m/z | 165.091 m/z | 5.45 | 3 | Sigma-Aldrich (Buchs, Switzerland) |
| Prostaglandin A2 | 9.93 | 10.6 | 317.210 m/z (-H_2_O) | 317.212 m/z (-H_2_O) | 6.305 | 3 | Adipogen  (Liestal, Switzerland) |
| Prostaglandin B2 | 9.93 | 10.8 | 317.210 m/z (-H_2_O) | 317.211 m/z (-H_2_O) | 3.152 | 3 |  |
| Prostaglandin J2 | 9.93 | 10.6 | 317.210 m/z (-H_2_O) | 317.212 m/z (-H_2_O) | 6.305 | 3 |  |

Abbreviation: RT, retention time
